# Supplementary material for: Advancing Personalized Medicine by Analytical Means: Selection of Three Metabolites That Allows Discrimination between Glaucoma, Diabetes, and Controls
Source: Metabolites. 2024 Feb 29;14(3):149. doi: 10.3390/metabo14030149 (PMC10971991; doi:10.3390/metabo14030149)
Supplement: Supplementary file 1 [file metabolites-14-00149-s001.zip › metabolites-2831247-supplementary.pdf]

metabolites

SUPPLEMENTARY MATERIAL

Advancing Personalized Medicine by Analytical Means: Selection of Three Metabolites That Allows Discrimination between Glaucoma, Diabetes, and Controls

David Bernal-Casas [1], Joan Serrano-Marín [2], Juan Sánchez-Navés [3], Josep M. Oller [1] and Rafael Franco [2,4,5,*]

[1] Department of Genetics, Microbiology and Statistics, Faculty of Biology, University of, 08028 Barcelona, Spain; bernalcd@ub.edu (D.B.-C.); joller@ub.edu (J.M.O.)
[2] Department of Biochemistry and Molecular Biomedicine, Faculty of Biology, University of Barcelona, 08028 Barcelona, Spain; joan.serrano.marin@ub.edu
[3] Department of Ophthalmology, Ophthalmedic and I.P.O, Institute of Ophthalmology, 07011 Palma de Mallorca, Spain; juansanchez.naves@gmail.com
[4] CiberNed, Network Centre for Neurodegenerative Diseases, National Spanish Health Institute Carlos III, 28029 Madrid, Spain
[5] School of Chemistry, University of Barcelona, 08028 Barcelona, Spain
* Correspondence: rfranco123@gmail.com or rfranco@ub.edu

Citation: Bernal-Casas, D.; Serrano-Marín, J.; Sánchez-Navés, J.; Oller, J.M.; Franco, R. Advancing Personalized Medicine by Analytical Means: Selection of Three Metabolites that Allows Discrimination between Glaucoma, Diabetes, and Controls. *Metabolites* **2024**, 14, 149. https://doi.org/10.3390/metabo14030149

Academic Editor: Joana Pinto

Received: 2 January 2024
Revised: 15 February 2024
Accepted: 23 February 2024
Published: 29 February 2024

Copyright: © 2024 by the authors. Licensee MDPI, Basel, Switzerland. This article is an open access article distributed under the terms and conditions of the Creative Commons Attribution (CC BY) license (https://creativecommons.org/licenses/by/4.0/).

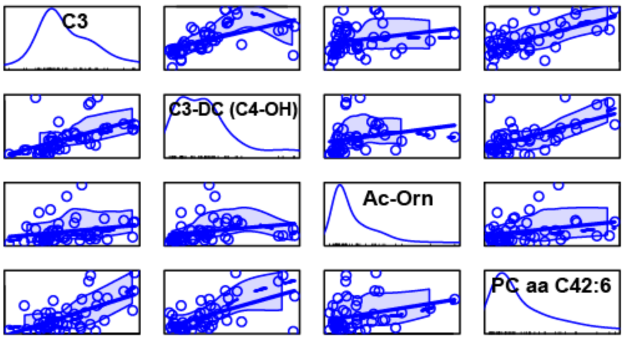

Supplementary Figure S1. Correlations between pairs of the four indicated metabolites. Values of one metabolite appear on the horizontal axis, and values of the other metabolite appear on the vertical axis. Each subject in the data appears as a point.

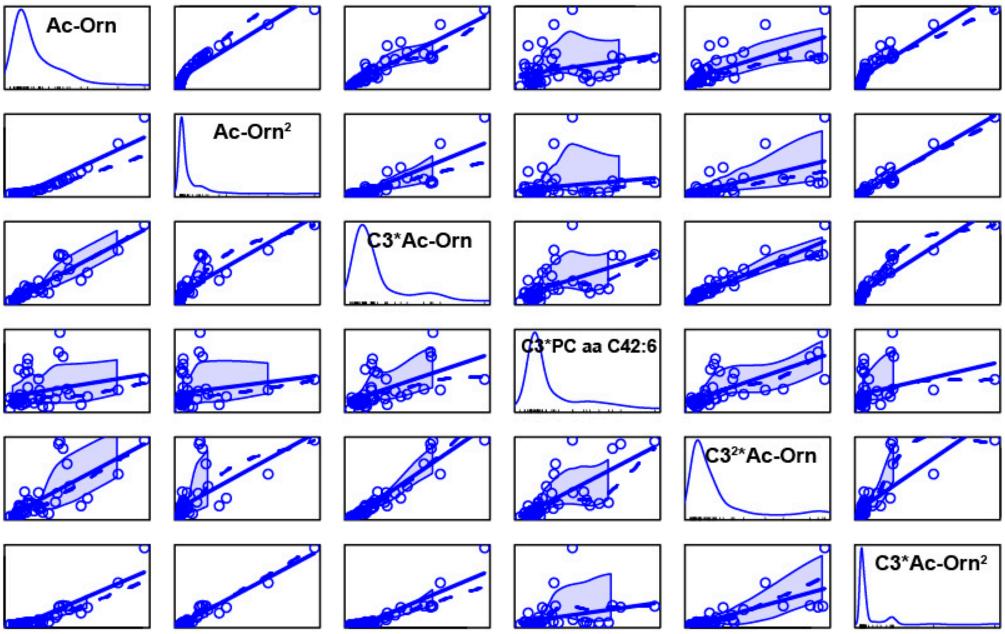

Supplementary Figure S2. Correlations between pairs of the six indicated variables. Values of one variable appear on the horizontal axis, and values of the other variable appear on the vertical axis. Each subject in the data appears as a point.
